# Clinical outcomes of digital sensor alerting systems in remote monitoring: a systematic review and meta-analysis

**DOI:** 10.1038/s41746-020-00378-0

**Published:** 2021-01-08

**Authors:** Fahad M. Iqbal, Kyle Lam, Meera Joshi, Sadia Khan, Hutan Ashrafian, Ara Darzi

**Affiliations:** 1 Division of Surgery, Imperial College London, St. Mary’s Hospital, London, W2 1NY UK; 2 Institute of Global Health Innovation, Imperial College London Faculty Building, South Kensington Campus, Kensington, London, SW7 2AZ UK; 3 Division of Cardiology, West Middlesex University Hospital, London, TW7 6AF UK

**Keywords:** Outcomes research, Health policy

## Abstract

Advances in digital technologies have allowed remote monitoring and digital alerting systems to gain popularity. Despite this, limited evidence exists to substantiate claims that digital alerting can improve clinical outcomes. The aim of this study was to appraise the evidence on the clinical outcomes of digital alerting systems in remote monitoring through a systematic review and meta-analysis. A systematic literature search, with no language restrictions, was performed to identify studies evaluating healthcare outcomes of digital sensor alerting systems used in remote monitoring across all (medical and surgical) cohorts. The primary outcome was hospitalisation; secondary outcomes included hospital length of stay (LOS), mortality, emergency department and outpatient visits. Standard, pooled hazard ratio and proportion of means meta-analyses were performed. A total of 33 studies met the eligibility criteria; of which, 23 allowed for a meta-analysis. A 9.6% mean decrease in hospitalisation favouring digital alerting systems from a pooled random effects analysis was noted. However, pooled weighted mean differences and hazard ratios did not reproduce this finding. Digital alerting reduced hospital LOS by a mean difference of 1.043 days. A 3% mean decrease in all-cause mortality from digital alerting systems was noted. There was no benefit of digital alerting with respect to emergency department or outpatient visits. Digital alerts can considerably reduce hospitalisation and length of stay for certain cohorts in remote monitoring. Further research is required to confirm these findings and trial different alerting protocols to understand optimal alerting to guide future widespread implementation.

## Introduction

With our ever-ageing population, a result of significant improvements in healthcare delivery, medicine, personal & environmental hygiene, a greater burden is placed on our primary and secondary care healthcare facilities^1^. The rising costs of healthcare delivery require novel strategies to improve healthcare service provision^2^, particularly one that proves to be cost-effective and is widely accepted by citizens.

Telemedicine, a concept since the 1970s, has evolved to be synonymous with terms such as digital health, e-health, m-health, wireless health, and, remote monitoring, among others. Indeed, over 100 unique definitions have been uncovered for ‘telemedicine’, a variation, which is likely to be attributed to the progression of these technologies^3,4^. Remote monitoring allows people to continue living at home rather than in expensive hospital facilities through the use of non-invasive digital technologies (such as wearable sensors) to collect health data, support health provider assessment and clinical decision making^5^. Several randomised trials have demonstrated the potential for remote monitoring in reducing in-hospital visits, time required for patient follow-up, and hospital costs in individuals fitted with cardiovascular implantable electronic devices^6–8^.

Vital signs including, heart rate (HR), respiratory rate (RR), blood pressure (BP), temperature, and oxygen saturations, are considered a basic component of clinical care and an aide in detecting clinical deterioration; changes in these parameters may occur several hours prior to an adverse event^9,10^. With wearable sensors being light-weight, small, and discrete they can be powerful diagnostic tools for continuously monitoring important physiological signs and offer a non-invasive, unobtrusive opportunity for sensor alerting systems to remotely monitor patients, driving the potential to improve timeliness of care and health-related outcomes^11^.

Feedback loops and alerting mechanisms allow for appropriate action following recognition of clinical deterioration. Current alerting mechanisms for remote monitoring include alert transmission to a mobile device; automated emails generated to a healthcare professional; video consultation; interactive voice responses; or web-based consultations^12^. The feedback loops can be relayed to nurses, pharmacists, physicians, counsellors, and physicians but also to patients^13^. Earlier recognition of deterioration, through alerting mechanisms, has potential to improve clinical outcomes, such as hospitalisation, length of stay, mortality, and subsequent hospital visits, through earlier detection but has been inadequately studied.

A recent systematic review reported outcomes for remote monitoring undertaken in individuals in the community with chronic diseases (e.g., hypertension, obesity, and heart failure), but many of the included studies were of low quality and underpowered; the meta-analyses were on obesity related intervention outcomes (body mass index, weight, waist circumference, body fat percentage, systolic blood pressure, and diastolic blood pressure), consisting of few studies^13^. Additionally, the evaluation of feedback loops and alerting mechanisms following recognition of abnormal parameters was not the main focus of this study, a pivotal phase where intervention could influence clinical outcomes. With the search performed in 2006, and the rapid evolution of such a field, an updated systematic review aimed at digital alerting mechanisms is warranted, with the inclusion of wider medical and surgical cohorts for generalisability. The aim of this systematic review is to identify studies evaluating digital alerting systems used in remote monitoring and describe the associated clinical outcomes.

## Results

### Study characteristics

**Fig. 1:**
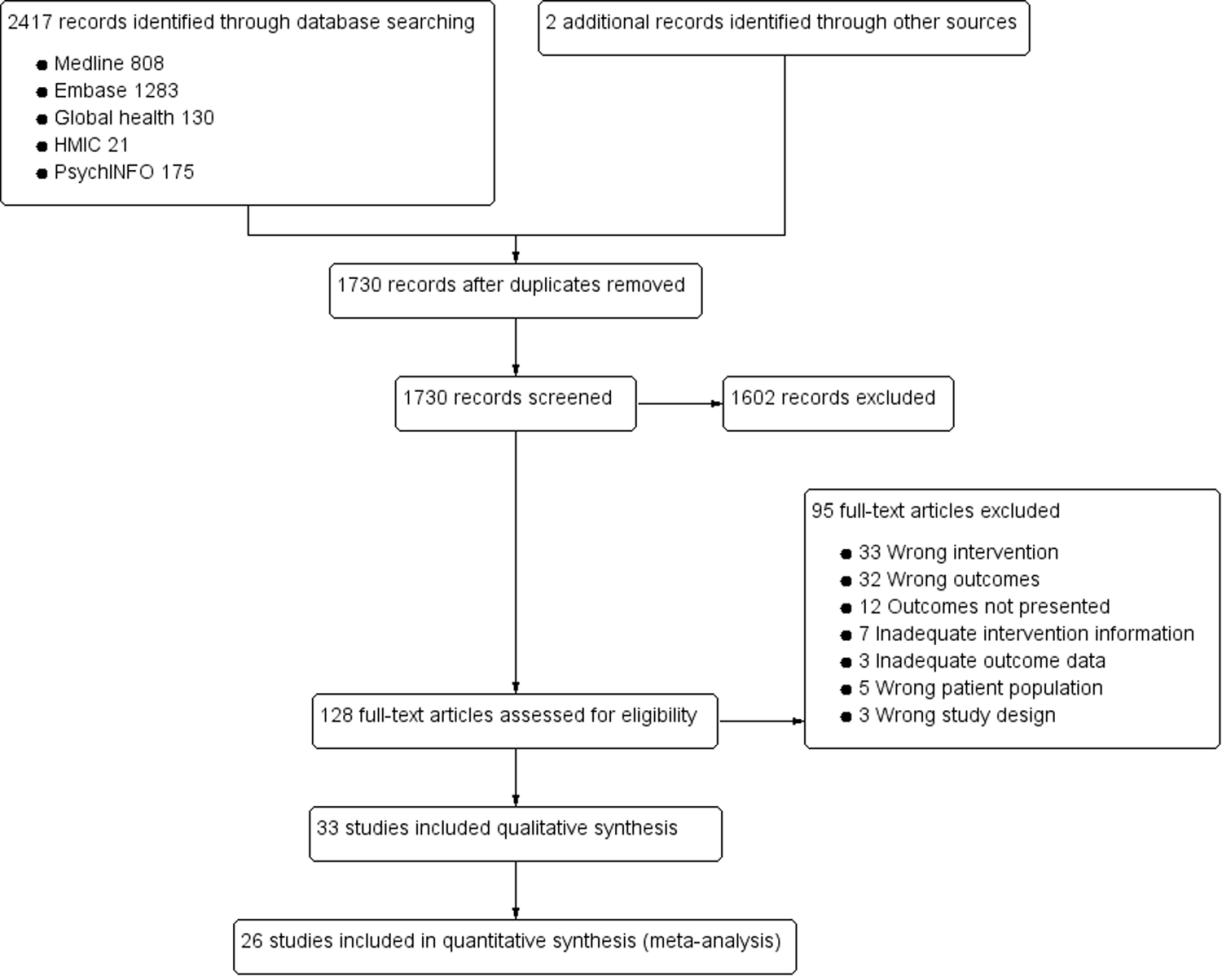
PRISMA flow diagram. Search and study selection process for this review.

A total of 2417 citations were retrieved through literature searches. An additional two articles were found from bibliography cross-referencing. Full-text review was performed for 128 articles with 33 meeting the inclusion criteria for analysis, of which, 21 were randomised controlled trials with the remaining prospective or retrospective studies. Of the 33 included studies, 23 allowed for meta-analysis. The characteristics of included studies is shown in Table 1. A PRISMA flow diagram can be seen in Fig. 1.

**Table 1:** Characteristics of included studies with quality score (Jadad & Newcastle-Ottawa Scale).

Author	Year	Title	Journal	Design	*N*	Follow-up	Score
Baker et al.^26^	2013	Effects of care management and telehealth: A longitudinal analysis using medicare data	*J Am Geriat Soc*	Retrospective	3534	2 years	High
Basch et al.^35^	2016	Symptom monitoring with patient-reported outcomes during routine cancer treatment: A randomised controlled trial	*J Clin Oncol*	RCT	766	6 months for quality of life; 12 months for mortality	Low^a^
Bekelman et al.^36^	2015	Primary Results of the Patient-Centred Disease Management (PCDM) for Heart Failure Study: A Randomised Clinical Trial	*JAMA Intern Med*	RCT	384	12 months	Low^a^
Biddiss et al.^55^	2009	Predicting need for intervention in individuals with congestive heart failure using a home-based telecare system	*J Telemed Telecare*	Prospective	45	18 (5) months (average, SD)	Moderate
Bohm et al.^20^	2016	Fluid status telemedicine alerts for heart failure: a randomised controlled trial	*Eur Heart J*	RCT	1002	18 months	Low^a^
Calvo et al.^37^	2014	A home telehealth programme for patients with severe COPD: The PROMETE study	*Respir Med*	Cluster RCT	59	7 months	Low^a^
Chen et al.^14^	2013	Clinical outcome and cost-effectiveness of a synchronous telehealth service for seniors and nonseniors with cardiovascular diseases: quasi-experimental study	*J Med Internet Res*	Prospective	141	6 months before and after	Moderate
Del Hoyo et al.^33^	2018	A Web-Based Telemanagement System for Improving Disease Activity and Quality of Life in Patients With Complex Inflammatory Bowel Disease: Pilot Randomised Controlled Trial	*J Med Internet Res*	RCT	63	24 weeks	Low^a^
Denis et al.^56^	2019	Prospective study of a web-mediated management of febrile neutropenia related to chemotherapy (Bioconnect)	*Support Care Cancer*	Prospective	41	3 weeks	Moderate
Godleski et al.^45^	2012	Home telemental health implementation and outcomes using electronic messaging	*J Telemed Telecare*	Prospective	76	6 months before and after	Moderate
Heidbuchel et al.^15^	2015	EuroEco (European Health Economic Trial on Home Monitoring in ICD Patients): A provider perspective in five European countries on costs and net financial impact of follow-up with or without remote monitoring	*Eur Heart J*	RCT	303	24 (±2) months	Low^a^
Kotooka et al.^21^	2018	The first multicenter, randomised, controlled trial of home telemonitoring for Japanese patients with heart failure: home telemonitoring study for patients with heart failure (HOMES-HF)	*Heart Vessels*	RCT	181	15 (0–31) months (mean, range)	Low^a^
Lee et al.^38^	2019	Telemedicine-Based Remote Home Monitoring After Liver Transplantation: Results of a Randomised Prospective Trial	*Ann Surg*	RCT	100	90 days	Low^a^
Lewis et al.^28^	2010	Does home telemonitoring after pulmonary rehabilitation reduce healthcare use in optimised COPD a pilot randomised trial	*COPD*	RCT	40	26 weeks telemonitoring + 26 weeks without (total 52 weeks)	Low^a^
Licskai et al.^46^	2013	Development and pilot testing of a mobile health solution for asthma self-management: asthma action plan smartphone application pilot study	*Can Respir J*	Prospective	22	3 months before and after	Moderate
Luthje et al.^22^	2015	A randomised study of remote monitoring and fluid monitoring for the management of patients with implanted cardiac arrhythmia devices	*Europace*	RCT	176	15 months	Low^a^
Martin-Lesende et al.^16^	2017	Telemonitoring in-home complex chronic patients from primary care in routine clinical practice: Impact on healthcare resources use	*Eur J Gen Pract*	Prospective	28	12 months before and after	Moderate
McElroy et al.^29^	2016	Use of digital health kits to reduce readmission after cardiac surgery	*J Surg Res*	Prospective	443	30 days	Moderate
Mousa et al.^34^	2019	Results of Telehealth Electronic Monitoring for Post Discharge Complications and Surgical Site Infections following Arterial Revascularization with Groin Incision	*Ann Vasc Surg*	RCT	30	30 days	Low^a^
Oeff et al.^47^	2005	[Monitoring multiple cardiovascular parameters using telemedicine in patients with chronic heart failure]	*Herzschrittmacherther Elektrophysiol*	Prospective	24	12 months before and after	Moderate
Pedone et al.^23^	2015	Efficacy of a Physician-Led Multiparametric Telemonitoring System in Very Old Adults with Heart Failure	*J Am Geriat Soc*	RCT	90	6 months	Low^a^
Pinnock et al.^30^	2013	Effectiveness of telemonitoring integrated into existing clinical services on hospital admission for exacerbation of chronic obstructive pulmonary disease: Researcher blind, multicentre, randomised controlled trial	*BMJ*	RCT	256	12 months	Low^a^
Pinto et al.^48^	2010	Home telemonitoring of non-invasive ventilation decreases healthcare utilisation in a prospective controlled trial of patients with amyotrophic lateral sclerosis	*J Neurol Neurosurg Psychiatry*	RCT	39	3 years	Low^a^
Ringbaek et al.^17^	2015	Effect of telehealthcare on exacerbations and hospital admissions in patients with chronic obstructive pulmonary disease: a randomised clinical trial	*Int J Chron Obstruct Pulmon Dis*	RCT	281	6 months	Low^a^
Santini et al.^49^	2009	Remote monitoring of patients with biventricular defibrillators through the CareLink system improves clinical management of arrhythmias and heart failure episodes	*J Interv Card Electr*	Prospective	67	11 (6–20) months (median, range)	Moderate
Scherr et al.^24^	2009	Effect of home-based telemonitoring using mobile phone technology on the outcome of heart failure patients after an episode of acute decompensation: randomised controlled trial	*J Med Internet Res*	RCT	108	6 months	Low^a^
Seto et al.^18^	2012	Mobile phone-based telemonitoring for heart failure management: A randomised controlled trial	*J Med Internet Res*	RCT	100	6 months	Low^a^
Sink et al.^39^	2018	Effectiveness of a novel, automated telephone intervention on time to hospitalisation in patients with COPD: A randomised controlled trial	*J Telemed Telecare*	RCT	168	8 months	High^a^
Smeets et al.^25^	2017	Bioimpedance Alerts from Cardiovascular Implantable Electronic Devices: Observational Study of Diagnostic Relevance and Clinical Outcomes	*J Med Internet Res*	Prospective	282	34 months (mean)	High
Steventon et al.^32^	2012	Effect of telehealth on use of secondary care and mortality: Findings from the Whole System Demonstrator cluster randomised trial	*BMJ*	Cluster RCT	3154	12 months	Low^a^
Steventon et al.^27^	2016	Effect of telehealth on hospital utilisation and mortality in routine clinical practice: A matched control cohort study in an early adopter site	*BMJ Open*	Retrospective	1432	10.4 months (average)	High
Vianello et al.^31^	2016	Home telemonitoring for patients with acute exacerbation of chronic obstructive pulmonary disease: a randomised controlled trial	*BMC Pulm Med*	RCT	334	12 months	Low^a^
Yount et al.^19^	2014	A randomised trial of weekly symptom telemonitoring in advanced lung cancer	*J Pain Symptom Manage*	RCT	253	12 weeks	Low^a^

*RCT* randomised controlled trial.

^a^Jadad scale.

### Hospitalisation and inpatient admissions

**Fig. 2:**
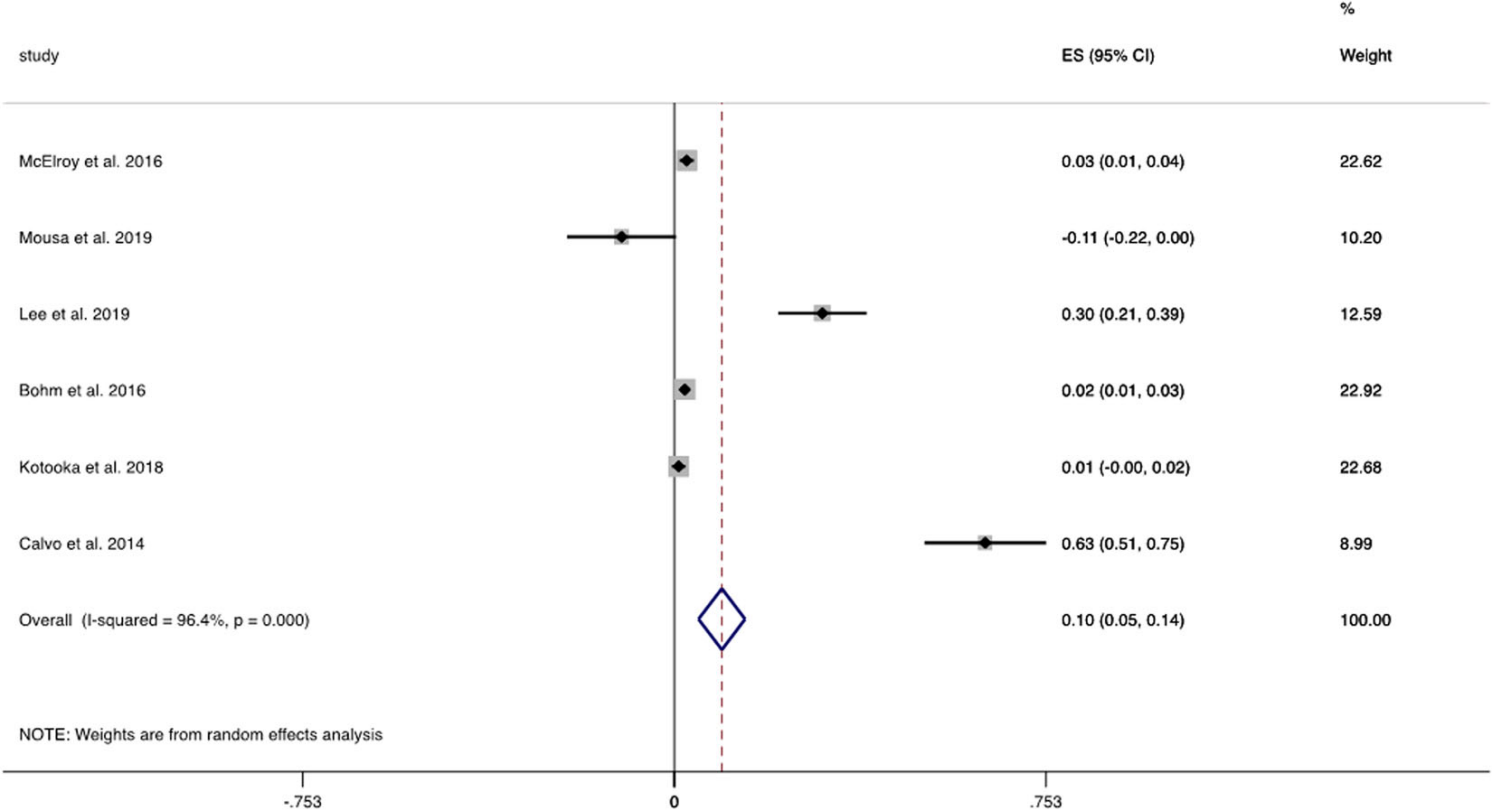
Forest plot hospitalisation. Forest plot of studies reporting hospitalisation and inpatient admissions.

Six studies demonstrated a mean decrease in hospitalisation/inpatient admissions of 9.6% (95% CI 4.9–14.3%, *I*^2^ = 96.4%, Fig. 2) favouring digital alerting systems from a pooled random effects analysis. However, pooled WMD reported no change in hospitalisation from six studies (WMD 0.061; 95% CI −0.197–0.318, *I*^2^ = 78%)^14–19^. Pooled HRs for all-cause hospitalisation similarly demonstrated no significant difference (HR 0.916; 95% CI 0.781–1.074, *I*^2^ = 0%)^20,21^.

Six additional studies, reporting on cardiovascular related hospitalisation, revealed no significant relationship with digital alerting (mean decrease 10.1%; 95% CI −24.9–4.7%, *I*^2^ = 95.6% and pooled HRs 0.907; 95% CI 0.757–1.088, *I*^2^ = 2.4%)^20,22–25^

### Mortality

**Fig. 3:**
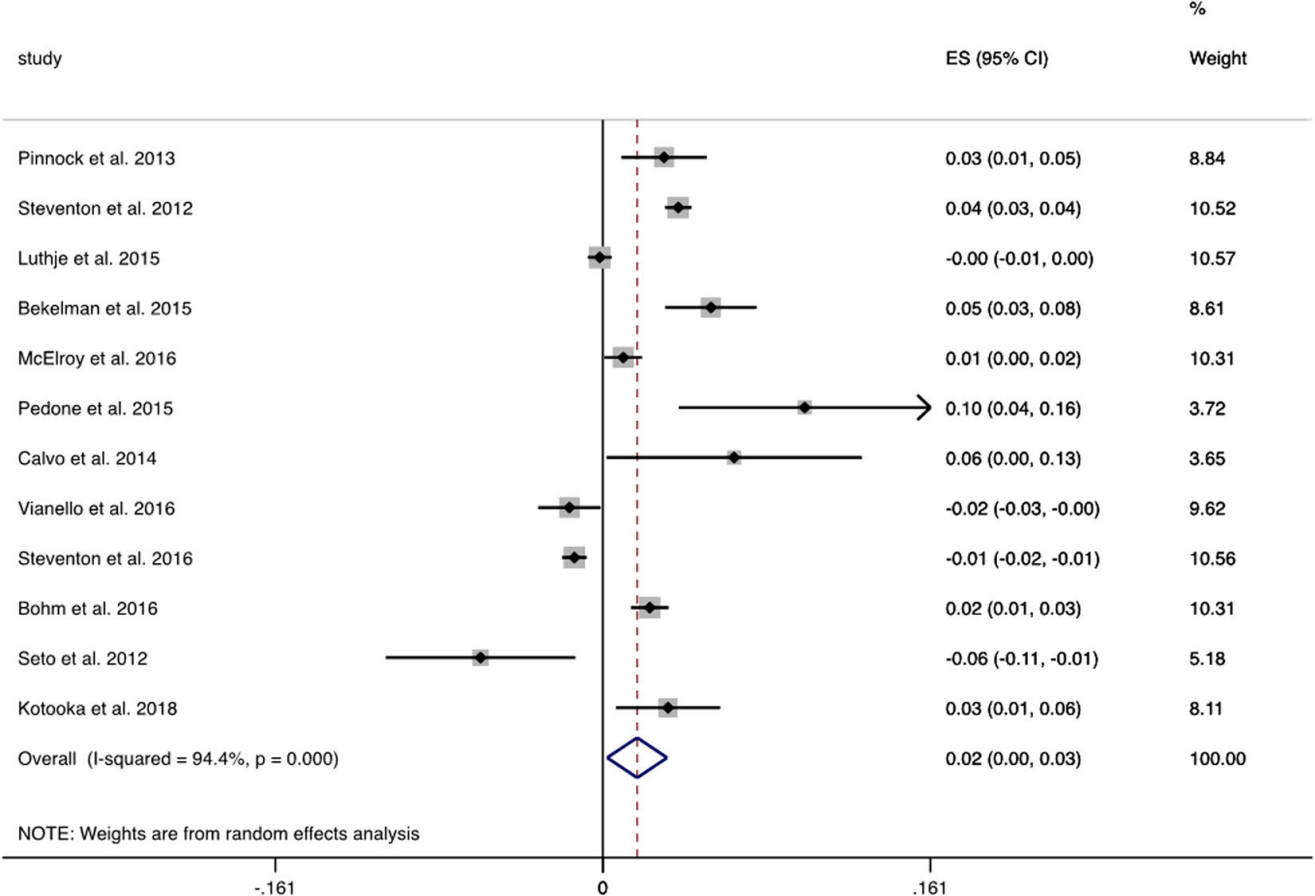
Forest plot mortality. Forest plot of studies reporting all-cause mortality.

A total of 16 papers were included; pooled random effects analysis demonstrated a 3% mean decrease in all-cause mortality from digital alerting systems (95% CI 2–3%, Fig. 3) from 12 studies; there was high heterogeneity with this analysis (*I*^2^ = 94.4%). However, pooled HRs of five studies reported no change in all-cause mortality (HR 0.89; 95% CI 0.79–1.01, *I*^2^ = 30.3%)^20,21,25–27^.

A sub-group cardiovascular cohort pooled random effect analysis failed to demonstrate a relationship between cardiovascular mortality and digital alerting (mean decrease 0.9%, 95% CI −0.6–2.4%, *I*^2^ = 25.7%)^20,24^.

### Length of stay

Ten studies were included; digital alerting reduced hospital LOS by a mean difference of 1.043 days (95% CI 0.028–2.058 days, *p* < 0.001, *I*^2^ = 95.5%)^14–18,24,28–31^. Three studies reported on LOS in chronic obstructive pulmonary disease (COPD) cases found no benefit of digital alerting (mean difference 0.919 days; 95% CI −1.878–3.717 days, *p* = 0.213, *I*^2^ = 35.3%)^17,30,31^.

### Emergency department visits

Eight studies were included; pooled random effects analysis of ED visits demonstrated no statistical benefit of digital alerting (mean difference 0.025; 95% CI −0.032–0.082, *I*^2^ = 51.8%)^14,16–19,22,28,32^.

### Outpatient and office visits

Five studies were included; pooled random effects analysis demonstrated no benefit of digital alerting (mean difference 0.223 days; 95% CI −0.412–0.858, *I*^2^ = 95.7%)^14,17,18,28,32^. Sub-group data from Ringbaek et al. (respiratory and non-respiratory) and Lewis et al. (primary care chest and non-chest related visits) were combined for this analysis.

Similarly, no statistically significant mean decrease in outpatient visits was noted from three additional studies^27,33,34^.

Sub-group analysis of a respiratory cohort demonstrated a mean difference of 1.346 days (95% CI 0.102–2.598, *I*^2^ = 93.8%)^17,28^.

### Risk of bias assessment

**Fig. 4:**
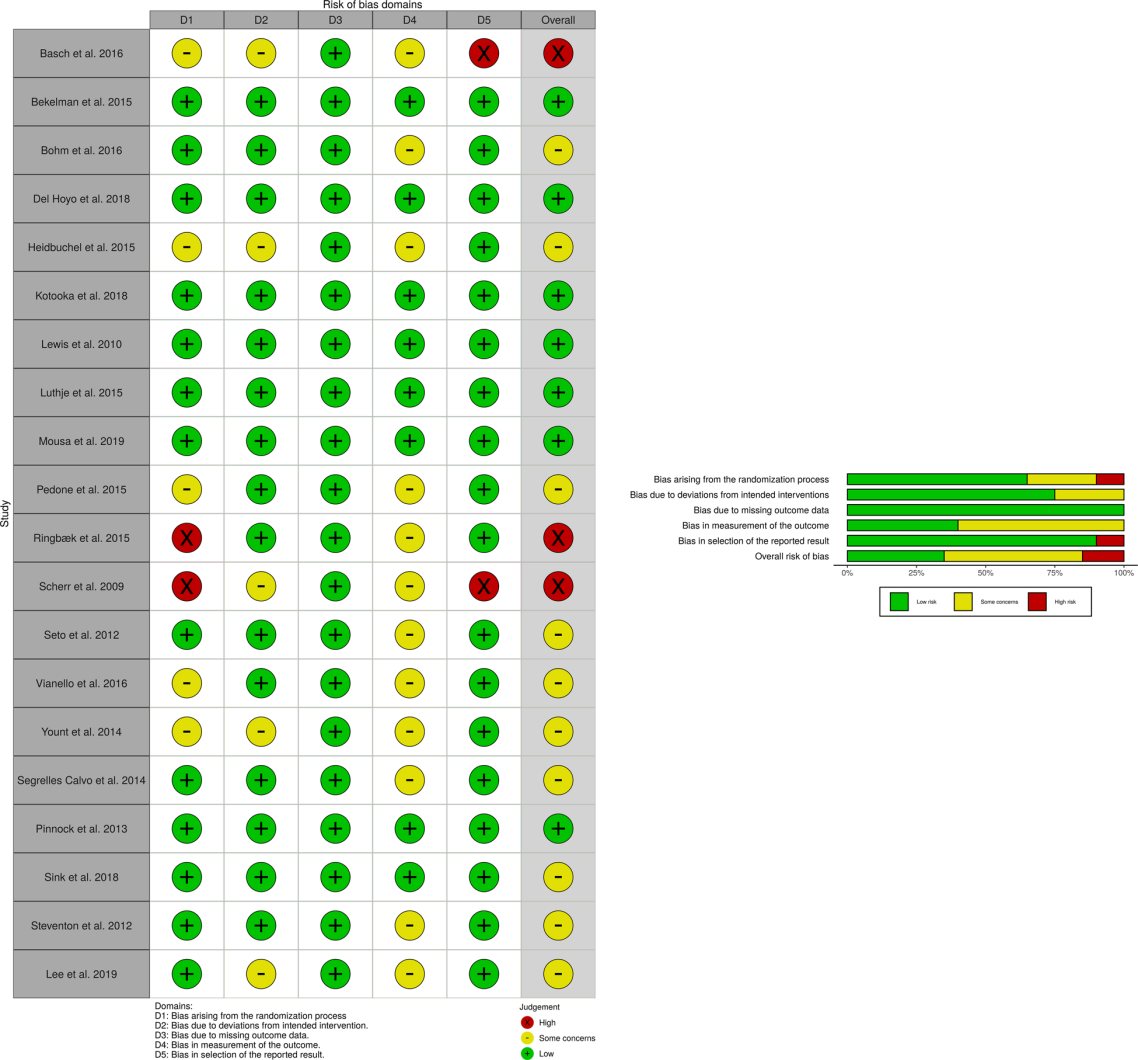
Risk of bias. Graphical display of the risk of bias results.

The assessment of risk of bias for included randomised trials is presented in Fig. 4.

Allocation was random across all 20 studies with 15 adequately stating the method used for generating random sequence^17–21,28,30,31,33–39^. Vianello et al.^31^ utilised a dedicated algorithm to check for imbalances for baseline variables with clear randomisation sequence methods detailed. However, concealment measures were not mentioned, resulting in a judgement of ‘some concerns’ for risk of bias for randomisation. Three additional studies were given the same judgement due to lack of concealment descriptions^15,19,35^. Ringbaek et al.^17^ clearly described their method for randomisation but information on concealment was not given and baseline demographic differences were noted between groups; as such, randomisation was judged to be at high risk of bias. Similarly, randomisation for Scherr et al.^24^ was deemed to be at high risk of bias.

Sink et al.^39^ blinded participants with digital alerts not forwarded to healthcare providers in the control arm. This, a result of their automated telephone intervention collecting self-reported symptom data rather than continuous physiological parameter recording through wearable sensors or smart devices, as utilised by the other trials, made participant blinding possible. A low risk of bias was, therefore, judged.

The risk of attrition bias was deemed low across all included studies with missing numbers clearly reported and deemed to not have impacted the overall results. There was mostly a complete follow-up of all participants.

Insufficient information was provided to assess whether other important risk of biases exists in four studies so were judged as some concerns^17,20,23,31^. Basch et al.^35^ clustered groups into computer experienced and computer in-experienced but numbers across various arms were unequal for selected outcome measures. Therefore, a judgement of high risk of bias was given. Comparably, Scherr et al.^24^ performed multiple analyses with both intention-to-treat and per-protocol. Only the latter revealed significant results favouring their telemonitoring system.

Overall, seven studies were deemed to be at low risk, ten studies had some concerns, and the remaining were judged as high risk of bias.

### Alerting mechanisms and response to alerts

Table 2 summaries the alerting mechanisms utilised within the studies. Mechanisms include text messaging, email notifications, alerts on telemonitoring hubs/web-based platforms, as well as, trialling audible alerts to study participants rather than healthcare professionals.

**Table 2:** Study characteristics of alerting mechanisms and responses.

Study	Cohort	Data collected	Digital alerting mechanism	Response to alerts	Control
Baker et al.^26^	HF; COPD; DM	Vital signs; symptom questionnaire; mental health questionnaire;	Health Buddy electronic device with four buttons to collect data and uploaded to a web-portal which risk stratifies responses.	Care manager review: specifics not mentioned.	Retrospectively matched
Basch et al.^35^	Oncology	Self-reported symptoms	Self-reporting through web-based interface (STAR). E-mail alerts triggered when a symptom worsened by >2 points or reached an absolute grade >3.	Nurses performed interventions: (1) telephone counselling, (2) medication changes, (3) Emergency/hospital referral)	Usual clinic visits with clinicians to discuss symptoms.
Bekelman et al.^36^	HF	BP; HR; weight; self-reported symptoms; mood	Daily telemonitoring using home-based equipment. The telemonitoring system assigned a risk to each response on the system.	Medium-risk indicators were reviewed by nurses for further action. All high-risk indicators were acted on by contacting the patient for assessment.	Usual care
Biddiss et al.^55^	HF	BP; HR; weight; quality of life questionnaire; symptom questionnaire	Biometrics entered daily into the ‘Doc@home’ health monitor. The data were transmitted at night through telephone. Alerts generated if pre-established thresholds crossed.	Monitoring practitioners contacted patient for further assessment.	-
Bohm et al.^20^	HF	Intrathoracic fluid status monitoring	OptiVol fluid index alert, changes in thoracic impedance resulting from accumulation of intrathoracic fluid generated a text message alert to responsible physician.	Data were reviewed remotely, and the patient contacted within 2 working days by phone to evaluate and take appropriate measures	Usual care without telemonitoring.
Calvo et al.^37^	COPD	Oxygen saturation; HR; BP; spirometry; peak expiratory flow	Daily monitoring of biometrics transferred through Tele-Modem™ to clinical monitoring team. A red alert was generated if pre-established thresholds were breeched in MPM™ call centre system.	A nurse contacted the patient to verify the alert. Following this, the alert was escalated to a Pneumologist. Actions include: (1) telephone advice, (2) home visits, (3) emergency department visits	Usual care
Chen et al.^14^	Coronary heart disease; HF; arrythmia; angina; syncope; DM	BP; HR; ECG; oxygen saturations; blood glucose	Real-time transmission of biometrics to health record clouds under synchronous surveillance by the Telehealth Centre. Alerting mechanism not specified.	Nurse case managers contacted the patient when abnormal data transmitted with advice ascertained from a cardiologist.	Pre-implementation
Del Hoyo et al.^33^	Inflammatory bowel disease	Weight; vital signs; quality of life	NOMHAD web-based home platform used. Electronic communication could take place between healthcare provider and users. Individualised alerts were generated for abnormal values.	After receiving an alert, the specialised medical staff, recommended action plans: (1) medication adjustment, (2) telephone calls, (3) in-person visits	Usual care in accordance with local and national guidelines.
Denis et al.^56^	Oncology	Temperature; symptom questionnaire	Bioconnect web application allowing daily biometric transmission. If algorithmic thresholds triggered, automatic email notifications were sent to the physician	Medical team called the patient for assessment. Actions include: (1) quick planned hospitalisation (bypass ED), (2) stay at home and blood test taken, (3) antibiotic administration	-
Godleski et al.^45^	Mental health	Symptom and behaviour questionnaire; substance abuse questionnaire	Health Buddy electronic messaging device used to answer questions daily by pressing large buttons on front of device. Nurse practitioner reviewed transmitted data and contacted the patient by telephone for concerning responses.	Actions included: (1) telephone assessment, (2) medication adjustment, (3) inpatient visit, (4) emergency department visit	Pre-implementation
Heidbuchel et al.^15^	CIED	CIED metrics	Continuous, automatic remote monitoring with frequency of data analysis and the response to alerts left to the investigator’s discretion.	Alerts resulting in: (1) hospital admissions, (2) internal discussions, (3) phone calls, (4) visits to physician, (5) web-review	Usual care (in office regular visits)
Kotooka et al.^21^	HF	Weight; BP; HR; body composition	Karada Karte™ telemonitoring system that transmitted data daily to the central web server via the internet. If pre-established parameter thresholds exceeded, monitoring nurses would notify the physician	Physician actions included: (1) telephone guidance, (2) medication changes, (3) warning threshold adjustment, (4) hospital admission	Usual care (in accordance with the 2010 Japanese Circulation Society Guidelines)
Lee et al.^38^	Transplant (liver)	Temperature; BP; blood glucose; weight; symptom questionnaire; medication use.	Tablet with bluetooth devices transmitted data daily to central web server via the internet. Different alerting algorithms trialled.	Alerts responded by the nurse care coordinator and escalated to care provider. Treatment or clinic visit initiated if appropriate.	Usual care: log vital signs daily for 90 days. Instructions provided for deterioration
Lewis et al.^28^	COPD	Temperature; oxygen saturations; HR; symptom questionnaire	Telemonitoring hub (Docobo™) transmitting biometrics to a web-based system (doc@HOME). An alerting e-mail was sent to the community team if pre-established thresholds were exceeded.	The chronic disease management team called patients on receipt of this alerting e-mail for further assessment during working hours (Mondays - Fridays, 9 a.m.–5 p.m.)	Usual care
Licskai et al.^46^	Asthma	Symptom questionnaire; peak expiratory flow; medication use.	The server analysed biophysical inputs daily. E-mail alerts were sent for moderate and high-risk days; and asthma control assessment displayed as green, yellow or red zone with the corresponding asthma management advice.	Asthma control assessment displayed as green yellow or red zone and gave appropriate asthma management advice.	Pre-implementation
Luthje et al.^22^	HF with CIED	Bioimpedance measurements from CIED	OptiVol fluid index alert, impedance value taken daily and compared with a roving reference value - built into the CIED.	Phone assessment with alerting patient was conducted. If signs of clinical decompensation, admit to hospital, if no signs of decompensation, adjust diuretic medication.	Usual care
Martin-Lesende et al.^16^	HF; chronic lung disease	BP; oxygen saturations; HR; RR; weight; symptom questionnaire	Daily self-monitoring of parameters sent using smartphones to a specific Web-platform. When pre-established threshold values were crossed, red or yellow alerts were triggered.	Not specified	Pre-implementation
McElroy et al.^29^	Cardiac surgery	Oxygen saturation; HR; BP; weight; symptom questionnaire; ambulation data; adherence to medication	Abnormal biometrics, concerning survey responses, missed digital check-ins registered through a digital health kit triggered an automated notification to the healthcare team.	Actions include: (1) video chat/phone call, (2) medication adjustment, (3) education, (4) referral to nurse practitioner/doctor/emergency department.	Discharge education booklet; medication education cards; interactive vital signs and weight log; phone call within 48 h of discharge and every 4–5 days for 30 days.
Mousa et al.^34^	Peripheral arterial disease (with groin incision)	Temperature; weight; BP; oxygen saturation; symptom questionnaire; surgical site pictures	Sensor metrics were uploaded to tablets with the Enform® application, syncing to a web-portal. Alerts were generated for values that exceeded pre-established thresholds.	Experienced nurses contacted patients by phone or used the app-integrated messaging for assessment following concerning alerts.	Usual care
Oeff et al.^47^	HF	Weight; BP; HR/rhythm; RR; oxygen saturations; symptom questionnaire	Daily telemonitoring transmission of biometrics. Alerts were generated when individualised limits were exceeded.	Actions include: (1) discussion with doctor; (2) medication adjustment; (3) planned hospital admission	Pre-implementation
Pedone et al.^23^	HF	BP; oxygen saturations; weight; HR	Geriatricians evaluated the data daily once transmitted through the telemonitoring kit. Alerts were generated if data exceeded an individualised prespecified range and were displayed on the monitoring system.	Actions taken: (1) scheduled office appointments, (2) acute care ward review	Usual care
Pinnock et al.^30^	COPD	Oxygen saturation; daily symptom questionnaire (dyspnoea, sputum purulence/volume, cough, wheeze, fever)	Algorithms, based on the symptom score, alerted the clinical monitoring team through secure internet connection, using a touch screen telemonitoring kit (Lothian), if daily readings had not been submitted daily or a certain score obtained.	Action include: (1) initiating patient contact. (2) home visit, (3) commencing rescue treatment, (4) immediate admission.	Usual care without telemonitoring
Pinto et al.^48^	Amyotrophic lateral sclerosis with respiratory failure on NIV	NIV data (IPAP, expiratory positive air pressure; inspiratory/expiratory ratio; backup rate; ventilation sensitivities; rise time	Data transmission with a modem through TCP/IP protocol occurred. All data that were SD ± 1 of the mean values of unpublished pilot data generated alerts.	A message was sent to the physician who could decide on possible setting changes, schedule an office visit or phone call, or conduct a real-time communication.	Management of NIV settings were performed through regular visits
Ringbaek et al.^17^	COPD	Spirometer; oxygen saturations; weight; self-reporting symptoms (dyspnoea, sputum colour/volume/purulence)	Data were transmitted daily to a call centre through telemonitoring equipment: categorised and prioritised with alerts generated if values were alarming.	Contact initiated by the respiratory nurse during working days (Monday–Friday, 9 a.m. to 3 p.m.).	Usual care
Santini et al.^49^	HF; arrythmias	Patient activity; HR and variability; intrathoracic impedance	Daily transmission through CareLink with an audible alarm to alert the patient when a programmable threshold is crossed.	If the patient was alerted or felt worse, to contact the responsible physician who request additional device transmissions, unscheduled visits or emergency room admissions.	-
Scherr et al.^24^	HF	BP; HR; weight; medication use	Data transmitted using a mobile telemonitoring kit (Zope) daily. Values outside individually adjustable borders resulted in an email/text alert.	Physicians contacted the patient directly via the mobile phone to confirm the parameters and adjust medication.	Usual care without telemonitoring
Seto et al.^18^	HF	Weight; BP; ECG; symptom questionnaires	Daily transmission of biometrics to a mobile phone, then transferred to a data repository. If pre-established thresholds crossed, email alerts sent to a cardiologist.	Dependent on cardiologist. Actions include retaking measurements, changing medication, attending emergency department or calling 911.	Usual care: visiting clinic between once every 2 weeks to once every 3–6 months.
Sink et al.^39^	COPD	Self-reported symptoms	Daily automated messages/calls daily from a central server to communicate disease-specific biometric data on ExpCOPD. The designed message algorithms use Bayesian branching logic to generate alerts to text, email, pager, or phone.	Following an alert, the medical resident contacted the patient for assessment and/or initiated appropriate intervention.	Received the same daily automated message without alerts.
Smeets et al.^25^	HF with CIED	Bioimpedance measurements from CIED	Daily alert transmissions generated when pre-defined alarm thresholds were crossed. OptiVol and CorVue algorithms for bioimpedance alerts generation.	Phone contact initiated by a nurse. Subsequent protocolised action was taken in consultation with a HF specialist.	CIED without bioimpedance alerts generated.
Steventon et al.^32^	COPD; HF	Oxygen saturations; blood glucose; weight; symptom questionnaires	Readings taken at the same time each day for up to 5 days per week, symptom questions and educational messages.	Monitoring centres (with specialist nurses and matrons), used protocolised responses.	Usual care
Steventon et al.^27^	COPD; HF; DM	Weight; oxygen saturation; BP; temperature; blood glucose; peak-flow; coagulation; 1-lead ECG	Readings taken and automatically transmitted to a triage centre through ‘mymedic’ telemonitoring hub.	If set thresholds were exceeded, patients were contacted; escalation to a physician for further plan was initiated.	Usual care
Vianello et al.^31^	COPD	HR; oxygen saturation	Alternate day recording of observations through a telemonitoring kit. Alerts generated when individualised pre-established thresholds crossed.	A pulmonary specialist called the patient for assessment during normal working hours (Monday–Friday, 0800–1600). Actions include: 1. Modify medication, 2. Home visit by district nurse, 3. Set up an office appointment, 4. Escalate a visit to the Emergency Department.	Usual care without telemonitoring
Yount et al.^19^	Advanced lung cancer	Symptom questionnaire	Weekly calls placed using telephone based interactive voice response system for symptom monitoring, responses entered using the telephone keypad.	Responses meeting a pre-defined threshold for a symptom generated an e-mail to the site nurse. Patients contacted for assessment.	Symptoms monitored weekly but no automated delivery

*COPD* chronic obstructive pulmonary disease, *HF* heart failure, *DM* diabetes mellitus, *CIED* cardiac implantable electronic device, *NIV* non-invasive ventilation, *HR* heart rate, *BP* blood pressure, *RR* respiratory rate, *STAR* Symptom Tracking and Reporting.

## Discussion

This meta-analysis provides evidence that digital alerting mechanisms used for remote monitoring are associated with reductions in hospitalisation and inpatient admissions. All pooled studies were prospective with the majority being randomised trials. However, most studies included were low in quality (Table 1) and only two studies had follow-up periods beyond 12 months^20,21^. The included studies were particularly heterogenous meaning that the results should be interpreted cautiously but may suggest that digital alerting in remote monitoring could be beneficial across a variety of patient cohorts. Pooled mean differences, however, did not reproduce this finding. The included studies consisted of longer follow-up periods^14–19^. One possible explanation could be that difference in cohorts analysed, with the latter containing more individuals suffering from chronic medical conditions (e.g., COPD, heart failure) compared to the former, which encompassed acute surgical cohorts with shorter follow-up periods.

A study in 2016 reported that avoidable hospitalisation increased by a factor of 1.35 for each additional chronic condition and 1.55 for each additional body system affected^40,41^. Clearly, a chronic disease cohort is particularly susceptible to recurrent hospitalisations and, while digitisation may play role in changing healthcare delivery, hospital departmental factors (e.g., seniority of clinician reviewing, busyness of department, community service delivery) and external factors (e.g., patient education and activation, behavioural insights towards digitisation, social support available) are likely to significantly contribute and may impact widespread deployment of novel digital technologies^42^.

Hospital length of stay was found to be reduced with digital alerting. This is likely a result of earlier recognition of deterioration resulting in prompt clinical review and treatment administration; a recent systematic review concluded that digital alerts similarly reduced hospital length of stay in sepsis by 1.3 days^43^. This review adds further support to the literature demonstrating the benefit of digital alerting in remote settings across medical and surgical cohorts.

A small reduction in all-cause mortality from digital alerting systems was noted. A relationship not reproduced from pooled hazard ratios, which may be explained by the difference of study qualities included in the analyses. Only three studies included were high quality; Of which, significant weighting was given to a 2013 study by Baker et al.^25–27^ utilising the Health Buddy telemonitoring platform, which has since become obsolete. Early iterations of digital alerting and telemonitoring platforms may suffer significant pitfalls, preventing successful use, a possible explanation for the described relationships.

Visits to the emergency departments demonstrated no benefit of digital alerting mechanisms from pooled mean differences. Earlier recognition of deterioration should prevent presentation to emergency departments and inpatient hospitalisations with non-urgent reviews scheduled for outpatient visits. Despite this, there was no change in overall outpatient or clinic visits. However, respiratory sub-group data did demonstrate a reduction in outpatient visits though the analysis was a culmination of only two studies. Further randomised trials for specific medical cohorts and conditions may address the benefit of digital alerting in affecting outpatient visits. Additionally, research capturing scheduled and unscheduled presentations to hospital, including emergency department visits, outpatient visits, and hospitalisations would be vital in addressing whether workloads can be altered across these departments.

Despite the significance of the outcomes assessed, our analysis had limitations based on the variety of methodologies used and overall study quality, with the majority scoring low. One of the challenges of this review was the relatively broad study into the effectiveness of digital alerting on clinical outcomes. While this allowed us to examine the similarities across various alerting mechanisms, it created significant heterogeneity. The justification of which was to determine effectiveness of alerting tools pragmatically across various cohorts, determining their overall efficacy as a tool to assist clinical decision making. Nevertheless, this limitation, largely a result of the paucity of high-quality literature, is to be acknowledged. The paucity in high quality, robust, literature limits the conclusions drawn in our review. The included non-randomised trials, due to their observational nature, are prone to selection biases, particularly pre-post implementation designs, which can be theoretically confounded by longitudinal changes in healthcare provision. Moreover, integrated feedback loops and responses to alerts are likely to feed into the Hawthorne effect^44^, an additional source of bias. Nonetheless, a great number of variables allowing for comprehensive characterisation of the digital alerting literature has been conducted which, to the authors’ knowledge, has not been undertaken previously.

Further research to answer several important questions is required. First, the optimal frequency of alerting; a range of remote monitoring schedules were utilised for data collection, including continuous^15^, daily^16–18,21–25,27,29,30,34,38,39,45–49^, only during office working hours (Monday–Friday)^28,31,32^, and weekly^19^. Indeed, given the diverse methodology in the literature, response time variation would be expected with potential for missing early signs of acute deterioration. Studies with less intense monitoring schedules may be suited for a cohort of individuals less prone to acute deterioration, regardless, a ‘window of opportunity’ presents itself for missing clinical deterioration in less frequent schedules. Second, which team members to be alerted and what nature of alert to be utilised. Alerts were frequently generated when pre-established thresholds, often tailorable, were breeched or for concerning responses to symptom questionnaires resulting in web-platform-based notifications, email alerts, telephone calls, texts, or pagers sent to members of a healthcare team (Table 2). In contrast, Santini et al.^49^ used audible alarms to alert patients when thresholds were breeched, empowering individuals to contact their responsible physician for further assessment. It is unlikely that one type of alert will be suitable for all individuals but further work identifying the most rapidly acknowledged and actionable alerts is required, including the exploration of alerts sent to individuals alongside healthcare professionals.

In conclusion, this review provides evidence that digital alerts used in remote monitoring can reduce hospital length of stay, mortality, and may reduce hospitalisations. Digital technologies continue to innovate and have the capacity to change current healthcare provision, particularly in the current COVID era. There is need for large, robust, multicentre, randomised trials studying digital alerting mechanisms in a varied cohort of individuals. Trials should seek to cycle different alerting protocols to understand optimal alerting to guide future widespread implementation not only within secondary and tertiary care settings but, importantly, in primary care, as implementation of new technologies within home settings has potential to truly revolutionise healthcare delivery.

## Methods

### Search strategy and databases

This systematic review was conducted in accordance to the Preferred Reporting Items for Systematic Reviews and Meta-analyses (PRISMA) guidelines^50^. The review was registered at the International Prospective Register of Systematic Reviews (PROSPERO ID: CRD42020171457).

A systematic search was performed using electronic databases through Ovid in Medline, EMBASE, Global health, health management information consortium (HMIC), and PsychINFO databases without language restriction. The appropriate MeSH terms and free text all field search was performed and combined with appropriate Boolean operators for “home”, “monitoring”, “remote sensing”, “self-monitor*”, “self-track*”, “remote monitor*”, “home monitor*”, “biosensing techniques”, “wireless technology”, “telemedicine”, “monitoring, physiologic”, “monitoring, ambulatory”, “home care services”, “ehealth”, “mhealth”, “telehealth”, “digital”, “mobile”, “social networking”, “internet”, “smartphone”, “cell phone”, “wearable electronic devices”, “internet”, “electronic alert*”, “alert*”, “messag*”, “text messaging”, “inform”, “communicat*”, “communication”, “patient-reported outcome measures”, “outcome and process assessment”, “outcome”, “treatment outcome”, “outcome assessment”, “fatal outcome”, “adverse outcome pathways”, “patient outcome assessment”, “morbidity”, “mortality”, “length of stay”, “patient admission”, “readmission”. Further studies not captured by the search were identified through bibliometric cross-referencing.

All identified studies were uploaded to Covidence, a Cochrane supported systematic review package tool^51^. Initial screening was conducted by one investigator and verified by a second to determine if the eligibility criteria were met. Discrepancies were discussed and resolved by consensus. Studies meeting the inclusion criteria underwent full-text screening; supplemental references were scrutinised for additional relevant articles.

### Study selection criteria and outcome measures

Studies published containing the primary and secondary outcomes listed below were included. No language restrictions were placed. Included study participants were adults (aged 18 years or over) discharged home with a digital alerting system (i.e., wearable sensor, non-invasive wireless technology, telemedicine, or remote monitoring). The last search was performed in October 2019.

Abstracts, conference articles, opinion pieces, editorials, case studies, reviews, and meta-analyses were excluded from the final review. Studies with inadequate published data relating to the primary and secondary outcome measures were additionally excluded.

### Data extraction

The primary outcome measure was hospitalisation and inpatient visits. Secondary outcome measures include mortality, hospital length of stay (LOS), emergency department visits, and outpatient visits.

All included study characteristics and outcome measures were extracted by one investigator and verified by a second. All full-text reports of studies identified as potentially eligible after title and abstract review were obtained for further review.

### Quality assessment (risk of bias)

Methodological quality of randomised trials (RCTs) was assessed with the Jadad Scale^52^. The scores range from 0–5; scores <3 were considered low quality and scores ≥3 were considered high quality^52^. The risk of bias Cochrane tool was used to assess internal validity; this assesses: (i) randomisation sequence allocation; (ii) allocation concealment; (iii) blinding; (iv) completeness of outcome data; and (v) selective outcome reporting, classifying studies into low, high or unclear risk of bias^53^. Non-randomised trials were assessed using the Newcastle-Ottawa scale^54^. It comprises three variables: (i) patient selection; (ii) comparability of study groups; and (iii) assessment of outcomes. Scores range from 0–9, scores ≤3 were considered low quality, between 4–6 moderate quality, and ≥7 high quality. Quality assessment was assessed by one reviewer and validated by a second.

### Data analysis

A standard, hazard ratio, and proportion of means meta-analyses were performed using Stata (v15.1. StataCorp LCC, TX). Effect sizes were transformed into a common metric (e.g., days for time). A percentage change for outcomes between control and intervention arms were calculated where possible. Hospitalisation and inpatient admissions were grouped into one variable.

Continuous variables were compared through weighted mean differences (WMD) with 95% CI. Where only the median was reported, it was substituted for mean. Where range was reported, it was converted to standard deviation through division of four. As assumption of normal distribution was made for this to occur. Forest plots were generated for all included studies.

Data were pooled using a random effects model and heterogeneity was assessed with the *I*^2^ statistic. We considered a value <30% as low heterogeneity, between 30 and 60% moderate, and over 60% as high.

## Data Availability

The datasets generated during and/or analysed during the current study are available from the corresponding author on reasonable request.
